# Spatio-Econometric Analysis of Urban Land Use Efficiency in China from the Perspective of Natural Resources Input and Undesirable Outputs: A Case Study of 287 Cities in China

**DOI:** 10.3390/ijerph17197297

**Published:** 2020-10-06

**Authors:** Xiao Han, Anlu Zhang, Yinying Cai

**Affiliations:** School of Public Administration, Huazhong Agricultural University, Wuhan 430070, China; hanvolks@webmail.hzau.edu.cn (X.H.); caiyinying@mail.hzau.edu.cn (Y.C.)

**Keywords:** urban land use, super efficiency SBM model, influencing factors, spatial econometrics

## Abstract

The rapid urbanization in China has had a huge impact on land use and the scarcity of land resources has become a constraint for sustainable urban development. As urban land is an indispensable material basis in economic development, measuring its use efficiency and adopting effective policies to improve urban land use efficiency (ULUE) are important links in maintaining sustainable economic growth. By establishing a comprehensive ULUE evaluation index system that emphasizes on incorporating the natural resources input and the undesirable output, ULUE from 2010 to 2016 was calculated based on super efficiency SBM model, and its potential influencing factors were explored using a spatial econometric model. The results show that: (1) temporally, the overall ULUE in China is upward growing, and the gap among regions is becoming gradually convergent. (2) Spatially, the ULUE of Chinese cities are positively correlated. (3) Economic agglomeration and industrial structure significantly improve ULUE in China, but the intensity of energy consumption has a negative impact on ULUE. We suggest that: (1) differentiated industrial development strategies should be formulated; (2) the economic growth pattern should be changed from energy-consuming to energy-saving; (3) priority should be given to innovation on science and education, so as to increase in clean energy input and cleaner production.

## 1. Introduction

Cities are increasingly becoming complex systems of social, economic and ecological subsystems [1], in which land plays an important role in urban production and life [2]. Human ever increasing demand driven by rapid urbanization exerts pressure on natural resources and induces consequent degradation of land quality and ecosystems [3], thereby reducing the high quality of urban development [4]. How to meet the requirement of high-quality urban development with limited land is becoming a global challenge [5]. Europe is a high-density populated area, where urban sprawl has encroached the suburban habitat, endangering the biodiversity. To address the challenges above, many countries in Europe such as Germany, France and The Netherlands have begun to adopt bio-economy strategies since 2002 [6,7]. Unlike the European Union (EU), North American is relatively sparsely populated and the main concerns with urban expansion are high-quality farmland loss and biodiversity disappearance. Both USA and Canada have tried zoning, green belts, urban growth boundaries and transferable development right systems to tackle unwanted urban growth [8]. The Latin American and South Asian regions have become rapid urban growth areas in the world since the 1970s, in which improperly planned urbanization led to urban slums, poor infrastructure development and encroachment of land property rights [9]. East Asia is the most densely populated region in the world, where skyrocketing housing prices, inadequate job opportunities and over conversion of farmland are common phenomena. Space for space in Japan, transferable development rights and block zone expropriation in Chinese Taiwan have been initiated to solve the above associated issues [10,11].

Compared with other countries in the world, China’s mainland urbanization has its own characteristics. Urbanization has lagged behind industrialization since the founding of the People Republic of China (PRC) for three decades [12]. Since China’s market-oriented reform and opening-up in late 1970s, China’s “the world manufacturing factory” economic strategy focused on heavy and low-end manufacturing such as petrochemical and textile products, which heavily rely on land and energy inputs [13]. The urban built-up area in China has soared from 7438 km^2^ in 1981 to 54,332 km^2^ in 2016, with an average annual growth rate of 5.68% [14]. The total energy consumption increased from 57.577 billion tons standard coal equivalent (SCE) in 1981 to 416.091 billion tons SCE in 2017, with an average annual growth rate of 5.65% [15]. In addition to high land and energy consumption, the rapid urbanization in China has also triggered the interactive coercion between resources and the eco-environment. Since the global economic crisis in 2008, the world economy has fallen into a period of long-term stagnation. Although the Chinese government unveiled a US $586 billion infrastructure package to restore economic growth through the creation of domestic demand, China’s GDP growth rate has continued to decline as the stimulus effect gradually dissipated. With weak economic growth, deteriorating environment, and intensified social conflicts, China may step into the “middle income trap” [16]. Hence, improving the utilization efficiency of limited urban land is an effective measure for alleviating the contradiction between environmental conservation and economic development.

Previous studies on ULUE can be traced back to the ecological school to visually identify the spatial structure of urban land use, mainly including axial mode, fan mode, concentric circle mode, etc [17]. Aiming at the disorderly sprawl of cities in developed countries, the concept of the compact city was proposed, which formed a new idea of efficient use of land and refined urban development from the aspects of compact function, compact scale and compact structure [18]. Since then, studies have focused on the definition, analysis and expansion of the concept of ULUE [19,20,21]. With the in-depth research, the connotation of ULUE shows a trend from structural efficiency, economic efficiency to comprehensive efficiency. 

An essential key to improving ULUE is to implement an effective evaluation approach [22]. The focus on ULUE evaluation is how to construct a scientific evaluation index system and select appropriate measurement methods. Following deepening understanding of the connotations of ULUE, the indicators for evaluation research have been gradually changing from a single indicator measurement system (such as “the urban economic output of each land unit”) to a multi-indicator measurement system [23,24]. ULUE evaluation methods have been evolving from descriptive- to quantitative-based models, mainly including cluster analysis [25], regression analysis [26], the SFA model [27] and the DEA method [28,29,30].

The analysis of influencing factors of ULUE contributes to further understanding of the causes and mechanisms of land use change, and then identifying countermeasures to improve ULUE [31]. From the perspective of influencing factors, studies generally focus on the following four aspects: economic factors [32,33], social factors [34,35], traffic factors [36,37] and political factors [38,39].

Although previous studies have made notable progress, there are some aspects to be improved: first, regardless of whether an undesirable output may overstate ULUE. The mainstream of ULUE studies consider a limited number of desirable land use outputs (e.g., economic benefits and social well-being) but ignore undesirable outputs (e.g., pollutants and industrial discharges during land use), which makes the research results incompatible with reality. Second, the neglect of natural resources inputs may overestimate the ULUE. The existing ULUE evaluation index systems do not account for the role of natural resources in the process of urban production activities and fail to reflect the depletion effect of economic development on the ecological environment. In fact, the value of natural resources is closely related to the sustainable development of the urban economy [40], because a certain proportion of urban production activities is at the cost of the rapid consumption of resources and the serious degradation of the ecological environment [41,42,43,44]. In 2005, the value of resources and environmental losses was US $408.29 billion, which means that 13.9% of China’s gross domestic product (GDP) was obtained at the expense of resource consumption, environmental pollution and ecological degradation [45]. These evidences imply that the lack of natural resources in the input indicator system will lead to artificially exaggerated land use benefits, which will overstate the efficiency. Third, the ignorance of the spatial effect may mislead the result. The classical regression model is mostly used to analyze the influencing factors of ULUE. However, due to factors such as industrial connections, trade exchanges, and technology diffusion among regions, the ULUE will be affected by spatial polarization effects or diffusion effects [46]. Therefore, empirical studies that ignored the spatial correlation could not manifest reliable results.

This study aims to estimate the comprehensive efficiency of urban land use and analyzes the influencing factors of ULUE. These specific objectives include three aspects: first, a comprehensive efficiency evaluation index system including economic and environmental factors is established. Second, the ULUE in 287 Chinese cities is calculated by using the super efficiency SBM model and the input saving potential analyzed according to the actual scale of inputs. Third, using spatial regression models, this study explores spatial correlation effects and influencing factors of ULUE. With the ULUE evaluation results and its influencing mechanism, the findings will provide a theoretic and practical foundation for optimized allocation, intensive use of urban land resources. 

The contribution of this research lies in two aspects: first, a comprehensive efficiency evaluation index system is established with appropriate consideration of natural resources input and undesirable output. Second, spatial autocorrelation of ULUE in China is recognized in the study of geographic space and spatial regression models have proven to be more useful in examining the influencing mechanism on ULUE.

The rest of the paper is organized as follows: The theoretical framework of this study is constructed in Section 2. Data and research methods are presented in Section 3. Section 4 provides the empirical results. Discussion and policy implications are given in Section 5. Finally, we draw the conclusions in Section 6.

## 2. Theoretical Analysis of New Type of Urbanization and ULUE

**Figure 1:**
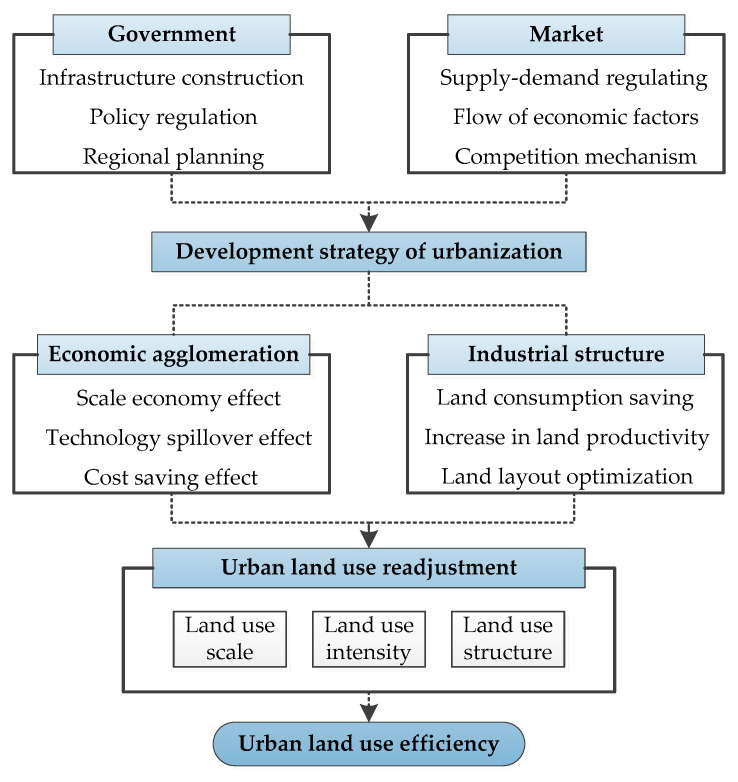
Theoretical analysis of new type of urbanization and ULUE.

In 2007, the “Development Strategy of New Urbanization (DSNU)” was promulgated by the Chinese government to abandon the extensive growth mode that mainly relies on investment expansion rather than productivity increases [47]. The DSNU sketched out a people-centered, eco-friendly development paradigm, which attempts to achieve the synergy development of environmental protection and economic growth [48]. The specific implementation path of DSNU is to create an institutional environment for the free flow of production factors through the reform of the administrative system and the improvement of the market mechanism. With the joint efforts of the government and the market, the scale and structure of urban industries will be continuously updated and adjusted. At the same time, due to the existence of increasing returns to scale and transportation costs, economic activities have agglomerated in self-evolution. Under the combined promotion of internal and external factors, the impact of changes in industrial structure and economic agglomeration will eventually be transmitted to other economic entities, thereby causing ULUE fluctuations. Therefore, under the above background, this study constructs an analysis framework that affects ULUE from the perspective of economic agglomeration and industrial structure (Figure 1).

### 2.1. Industrial Structure Transition and ULUE

The process of industrial structure adjustment reflects the changes in the level of social productivity, that is, the transfer of production factors from low-efficiency industries to high-efficiency industries [49]. Industrial structure transition affects the ULUE by changing land use pattern, intensity, and spatial layout. The impacts are mainly manifested in three aspects.

First, the transformation of industrial structure is conducive to saving land consumption. Urban land use in China is characterized with large-scale expansion of space and high proportion of industrial land. In 2018, inefficient urban land area accounted for 9.85% of the urban built-up area [50]. Driven by the substitution effect, land with relatively higher prices will be replaced by other input factors with lower prices. Besides, the traditional industries that use larger land areas will gradually be replaced by new industries that occupy less land. These two aspects will result in a significant reduction in actual land consumption.

Second, the industrial structure transition makes contribution to improving land returns. The changes in the industrial structure are mainly manifested in the technological intensification of the industrial structure, the accumulation of knowledge and economic servitization, the increase in industrial relevance, the extension of the length of the industrial chain, and the increase in industrial added value. In other words, optimizing the industrial structure, especially increasing the proportion of the tertiary industry and emerging industries, can increase the output efficiency of unit land area. 

Third, the optimization of the industrial structure will help to regulate the spatial allocation of land resources. Emerging industries (modern financial industry, advanced manufacturing and other industries) have strong ability to bid for rent [51]. Under the effect of the price mechanism, urban land can be allocated to industries with the highest willingness to pay, so that land resources can be optimally allocated spatially, which eventually transmitted to the improvement of ULUE.

### 2.2. Economic Agglomeration and ULUE

The impact of economic agglomeration on ULUE is realized through the agglomeration effect, which is specifically manifested in three aspects:

(1) Scale economy effect. The regional division of labor and regional differences can effectively promote the formation of economic agglomeration. Meanwhile, agglomeration will further strengthen the division of labor and specialization. They are sharing mutual promotion and common development. In this process, land users in the agglomeration area will gradually achieve the optimal level of economic scale, and can take the advantage of scale economy, which will greatly increase the land revenue [52].

(2) Technology spillover effect. Economic agglomeration provides convenient conditions for information and knowledge exchange between individuals and enterprises and realizes resource sharing and complementary advantages. Individuals or enterprises generate positive externalities of technology through their “learning effects” [53]. These spillover effects reduce the cost of enterprise research and development, stimulate the innovation vitality of enterprises, guide the enterprise to achieve technological progress and factor productivity through innovation, and promote the intensive and efficient use of land. 

(3) Cost saving effect. In the context of economic agglomeration, the gradual improvement of industry information transmission and service systems provides a prerequisite for the realization of services, infrastructure sharing and inter-industry communication. Therefore, land users can easily obtain market information and related services, thereby greatly reducing transaction costs, which are mainly reflected in management, financing and marketing.

However, it must be seen that when the degree of urban economic agglomeration continues to increase, congestion effect may occur. Once the congestion effect exceeds the agglomeration effect, urban agglomeration will cause the marginal output rate of land to continue to decrease, or even be negative, resulting in a corresponding decline in ULUE [54].

## 3. Methodology and Data Specification

**Figure 2:**
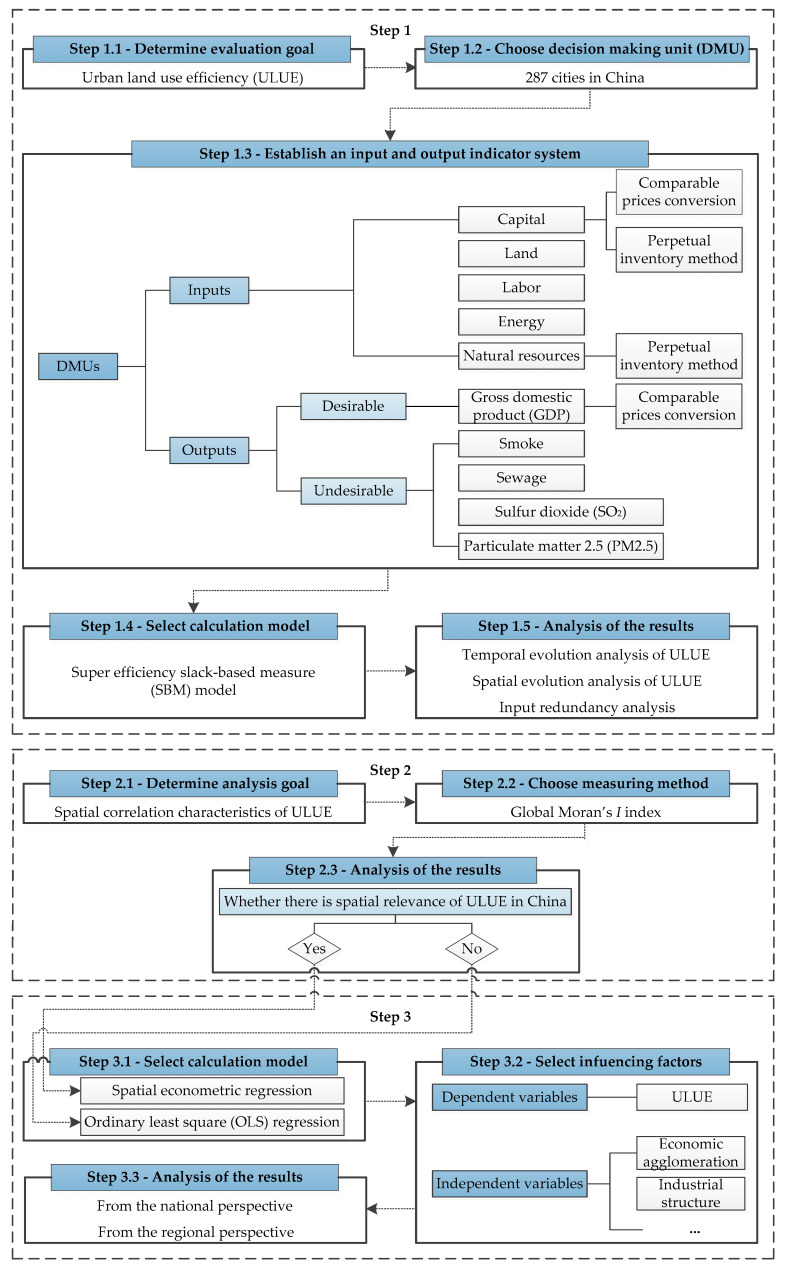
The flowchart of research.

In this study, we first measure the ULUE of 287 cities in China with a super efficiency SBM model (Step 1). Then the spatial autocorrelation analysis approach is employed to explore the spatial interaction mechanism of each research object over the study period (Step 2). After that, we use the spatial panel model to estimate the impacts of industrial structure and other factors on ULUE (Step 3). The flowchart of the research method is shown in Figure 2.

### 3.1. Research Area

The research area of this paper is 287 cities in China, excluding Hong Kong, Macao, and cities in Taiwan and Tibet because of the unavailability of the relevant data. Due to the internal differences among resource endowments and socioeconomic contexts in China, the sample cities are divided into the following four regions: eastern region, central region, western region and northeastern region. Among them, the eastern region includes Beijing, Tianjin, Shanghai, and cities in Hebei, Jiangsu, Zhejiang, Fujian, Shandong, Guangdong, and Hainan; the central region includes cities in Shanxi, Anhui, Jiangxi, Henan, Hubei, and Hunan; the western region includes Chongqing, and cites in Guangxi, Sichuan, and Guizhou, Yunnan, Shaanxi, Gansu, Qinghai, Ningxia, Xinjiang and Inner Mongolia; the northeastern region includes cities in Heilongjiang, Jilin, and Liaoning.

### 3.2. Indicator Selection

The method of indicator selection is primarily based on the principle of input vs. output during land use. The index system includes both inputs and outputs. As mentioned above, environmental and economic factors must be considered simultaneously when constructing the evaluation system [55,56]. According to the principle of representativeness and usability of indicators, this study constructs an indicator system for ULUE evaluation, the input and output indicators are described in Table 1.

(1) Input indicators

The land, labor, capital and energy are selected as input indicators, corresponding to industrial land, the amount of industrial labor, total investment in fixed assets and the annual energy consumption, respectively. At the same time, the natural resources input is included in the input elements to reflect the real consumption of ecological resources by regional economic development. In this paper, ecosystem service value is used to represent the input of natural resources. The calculation of ecosystem services value mainly refers to the research results of Xie et al. [57].

(2) Output indicators

We choose the added value of the secondary and tertiary industries as desirable output, and smoke emissions, sewage discharge, sulfur dioxide emissions and urban PM2.5 annual average index as four undesirable outputs.

### 3.3. Method for Measuring ULUE

Data envelop analysis (DEA) does not require any prior functions or parameter weights and it has become the most widely used method in efficiency evaluation [58,59]. The traditional DEA method considers a group of objects called decision making units (DMUs), weighing output factors and input factors to measure how close each DMU is to the effective state. However, the traditional radial DEA models neglect the slack variables in the objective function, which may produce inaccurate results. To overcome this problem, Tone [60] proposed a slack-based measure (SBM) model in 2002, a method based on slack variables to evaluate the efficiency of DMU. Compared with traditional DEA models, the SBM model directly incorporates slack variables into the objective function. The economic interpretation of the model is to maximize actual profits, not just the benefit ratio. The SBM model is described as follows:

(1) $$min\rho =\frac{1-\frac{1}{m}\sum_{i=1}^{m}s_{i}^{-}/x_{ik}}{1+\frac{1}{s_{1}+s_{2}}\left(\sum_{i=1}^{s_{1}}s_{i}^{g}/y_{ik}^{g}+\sum_{i=1}^{s_{2}}s_{i}^{b}/y_{ik}^{b}\right)}$$

$$s.t.\{\begin{array}{c} X\omega +S^{-}=x_{k}\\ Y^{g}\omega +S^{g}=y_{k}^{g}\\ Y^{b}\omega +S^{b}=y_{k}^{b} \end{array}$$

where ρ represents the value of ULUE. Suppose there are m DMUs, $S^{-}$, $S^{g}$, $S^{b}$ refer to slack variables of the DMUs’ input, desirable output and undesirable output, respectively. x, $y^{g}$, $y^{b}$ refer to the values of input, desirable output and undesirable output, respectively. m, $s_{1}$, refer to the numbers of input, desirable output and undesirable output, respectively. X, $Y^{g}$, $Y^{b}$ corresponds to the matrix composed of input, desirable output and undesirable output, respectively. ω is a weight vector. The objective function is monotonically decreasing, and 0 ≤ ρ* ≤ 1, when ρ* = 1, namely $S^{-}=0$, $S^{g}=0$, $S^{b}=0$, the DMU is effective. When ρ* < 1, the DMU is ineffective and needs to make input improvements. Whereas the BCC model is unable to differentiate DEA-efficient DMUs whose efficiency values equal 1, super-efficiency DEA can solve this problem. The idea is based on the first use of SBM evaluation for DMU, and then using super efficiency evaluation to evaluate the effective DMU [61].

### 3.4. Method for Exploring the Distribution and Temporal Dynamic Evolution of ULUE

Kernel Density Estimation (KDE) is an important method of non-parametric estimation. The KDE mainly views the distribution pattern of investigation objects as a probability distribution and then investigates the variation of its characteristics over time [62]. Distribution positions, forms, and ductility of ULUE can be known by graphic comparison of KDE results. The KDE function can be defined as:

(2) $$f\left(x\right)=\frac{1}{Nh}\overset{n}{\underset{i=1}{\sum }}K\left(\frac{x-x_{i}}{h}\right)$$

where K(·) is the kernel function, N is the number of observed values, h is the bandwidth, $x_{1},x_{2}\ldots ,\;x_{n}$ are samples from a continuous population x. In this paper, the Gaussian normal distribution kernel function is used:

(3) $$K\left(x\right)=\frac{1}{\sqrt{2\pi }}\exp\left(-\frac{x^{2}}{2}\right)$$

### 3.5. Method for Exploring the Spatial Correlation Effect of ULUE

Spatial autocorrelation analysis is an important method for exploring spatial interaction mechanism among research objects. Moran’s *I* index is a commonly used statistical index to measure the degree of spatial autocorrelation, in which the global autocorrelation Moran’s *I* index is used to consider the distribution characteristics of objects in the global space [63]. In this paper, it is used to diagnose whether there is a spatial correlation in ULUE. The global Moran’s *I* expression is:

(4) $$I=\frac{n\sum_{i=1}^{n}\sum_{j=1}^{n}w_{ij}\left(x_{i}-\overset{-}{x}\right)\left(x_{j}-\overset{-}{x}\right)}{\sum_{i=1}^{n}\sum_{j=1}^{n}w_{ij}\sum_{i=1}^{n}{\left(x_{i}-\overset{-}{x}\right)}^{2}}=\frac{\sum_{i=1}^{n}\sum_{j\neq i}^{n}w_{ij}\left(x_{i}-\bar{x}\right)\left(x_{j}-\bar{x}\right)}{S^{2}\sum_{i=1}^{n}\sum_{j\neq i}^{n}w_{ij}}$$

where I denotes global Moran’s *I* statistics of ULUE, n represents the number of study objects. the $x_{i}$ and $x_{j}$ are the ULUE values in city i and j, respectively. $\overset{-}{x}$ is the mean value of ULUE, $w_{ij}$ is the spatial weight matrix, denoting the spatial positional relationship between areas i and j. When i and j are adjacent spatial positions, $w_{ij}$=1; otherwise, $w_{ij}$=0. The value of global Moran’s *I* statistic is usually in the range of −1 to 1. A global Moran’s *I* value greater than 0 indicates positive correlation, and a value approximately equal to 1 indicates that similar attributes are agglomerated together. If the statistic is smaller than 0, it indicates negative correlation; if it is approximately equal to −1, it indicates that mutually exclusive attributes are agglomerated. A global Moran’s *I* approximately equal to 0 indicates that the attributes are randomly distributed or there is no spatial autocorrelation. 

### 3.6. Method for Spatial Influencing Analysis of ULUE 

The preconditions of the traditional econometric model are the spatial homogeneity and independent isomorphism of the sample. When applying the ordinary least squares (OLS) method for parameter simulation, the spatial correlation of the residual term is neglected, which tends to cause deviation of the results. Therefore, it is necessary to use spatial econometric models to solve the spatial dependence and correlation of the variables. The spatial regression models herein mainly include the spatial lag model (SLM) and the spatial error model (SEM). The SLM formulated as follows:

(5) $$\{\begin{array}{c} y_{ij}=\rho \overset{N}{\underset{j=1}{\sum }}w_{ij}y_{ij}+\beta X_{it}+\mu_{i}+\varepsilon_{it}\\ i=1,\ldots ,N\\ t=1,\ldots ,T \end{array}$$

where $y_{ij}$ stands for the ULUE for city i at time t; $\overset{N}{\underset{j=1}{\sum }}w_{ij}y_{ij}$ are the endogenous interaction effects of the $y_{ij}$; X is a NT×M matrix of the explanatory variables supposing that there are m variables. ρ is the spatial autoregressive coefficient which reflecting the influences of contemporaneous spatial correlation between one city and other geographically proximate cities; $w_{ij}$ is the spatial weight matrix of the form N×N dimensions. The term $\mu_{i}$ represents a spatial unit with the individual specific. $\varepsilon_{it}$ is an NT×1 random error vector, it should be unrestrictedly and equivalently distributed with a zero mean and variance $\left(0,\sigma^{2}\right)$.

When the interaction between regions differs due to the relative geographical space, the SEM is adopted, and the model is set as:

(6) $$\{\begin{array}{c} y_{ij}=\beta X_{it}+\mu_{i}+\varphi_{it}\\ \varphi_{it}=\lambda {\;}^{\prime }\overset{N}{\underset{j=1}{\sum }}w_{ij}\varphi_{ij}+\varepsilon_{it} \end{array}$$

where $\varphi_{it}\;$stands for the spatial autocorrelation error term. $\lambda {\;}^{\prime }$ represents the term of spatial autocorrelation coefficient. The other parameters are the same as mentioned above.

To select the most suitable spatial econometric model, several key procedures need to be further completed. First, the applicability of the SLM and the SEM will be judged by Lagrange multiplier (LM) and robust Lagrange multiplier (R-LM). Second, a Hausman test is used to choose between fixed-effect model and random-effect model. Finally, the optimal model will be selected from the time fixed, individual fixed and double fixed effect models according to the goodness of fit test.

Against the background of DSNU implementation, the dominant factors affecting ULUE are economic agglomeration and industrial structure. Besides, control variables such as urban economic development level and government intervention are added to the research model to reflect the influencing factors more comprehensively (Table 2). The economic agglomeration is measured by population density. The industrial structure is measured by the ratio of the output value of the tertiary industry to the secondary industry. The economic development is characterized by the per capita GDP of the sample cities. The government intervention is measured by the per capita fiscal expenditure. The investment in science and education (sci & edu) is expressed by the proportion of science and education expenditure to fiscal expenditure. The environmental governance capability is measured by a comprehensive rate calculated by three normalized indexes of exhaust gas treatment rate, waste water treatment rate and garbage disposal rate based on the entropy weight method. Energy consumption intensity is measured by unit GDP energy consumption. The land marketization is characterized by the ratio of the land area for bidding and auction to the total area of the transfer.

### 3.7. Data Sources

The data are mainly from public information sources including China Statistical Yearbook (2002–2015) (CSY), China Urban Construction Statistical Yearbook (2001–2014) (CUCSY) and China Statistical Yearbook on Environment (2002–2015) (CSYE). The land use data of this paper come from the land use cloud platform of the Ministry of Land and Resources, and the urban construction land data is reorganized according to the results of the national land use change survey. To eliminate the effects of price factors, urban investment in fixed assets and GDP value of secondary and tertiary industries are converted to 2010 constant prices with fixed assets and GDP deflectors, respectively. For the missing data, the interpolation method and the smooth growth method are used to obtain the supplementary value.

## 4. Results

### 4.1. Analysis of ULUE Results

#### 4.1.1. Spatio-Temporal Characteristics of ULUE

The Maxdea 7.0 software (Realworld Software Company Ltd., Beijing, China) is used to estimate the ULUE of 287 cities in China based on the super efficiency SBM model (Table 3). China’s average ULUE score, with a value of just 0.553, is consistently low during the research period, which indicates that the input and output conversion level of most cities in China is insufficient, and there is still great potential for ULUE improvement. During the years 2010 to 2016, China experienced a slow continuous improvement in ULUE. Specifically, the efficiency value increased by 14.2%, from 0.528 in 2010 to 0.603 in 2016. This implies that government has paid attention to the rationality of land use, which has shrunk the distance between actual production input and ideal marginal efficiency frontier.

**Figure 3:**
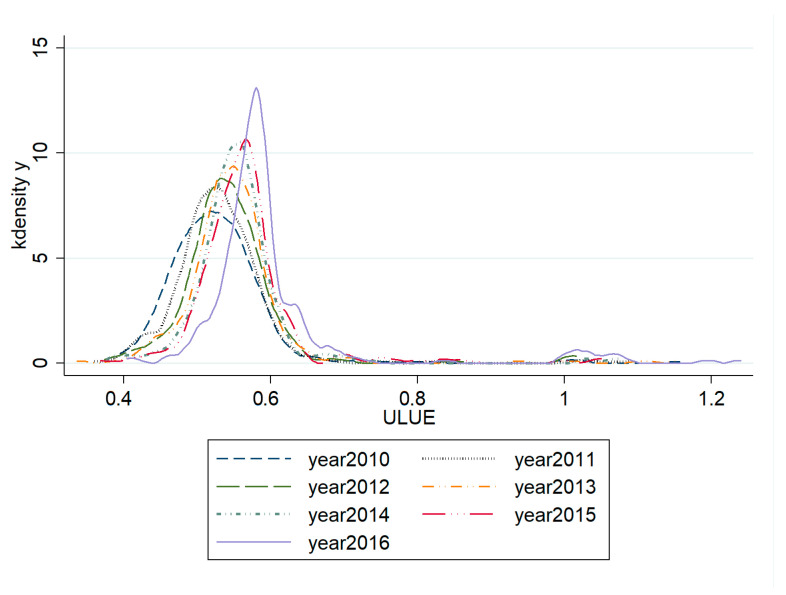
Kernel density estimation of ULUE in China during 2010–2016.

Figure 3 shows the KDE results of ULUE in the selected years. The horizontal axis represents the level of ULUE, and the vertical axis represents the kernel density. The kernel density map shows a large change in the shape of the skewed distribution of ULUE in China. Specifically, the curve is relatively smooth in 2010 and has the characteristic of a “long tail”, indicating that the polarization effect is weak, but the regional differences are large. From 2010 to 2016, the center of the curve continued to move to the right, the kernel density of the main peak increased significantly and the curve shape became steeper. This trend demonstrates that the degree of polarization effect has slowly increased, and the regional differences among cities have gradually narrowed. In addition, the kernel density curve shows a double peak in 2016 and the density of the main peak is much higher than that of the second peak, which suggests that ULUE should have significant multi-level differentiation.

From a regional perspective (Table 3), the level of ULUE is relatively high in eastern region, followed by western region, and low in central and northeastern regions. The average ULUE values of these four regions from 2010 to 2016 are 0.588, 0.532, 0.546 and 0.539, respectively. In the research time series, except for the ULUE value of the northeastern and eastern regions, which fluctuated in 2011 and 2013 respectively, the average ULUE in all regions showed an uptrend. Although the level of ULUE in the eastern region has always been at the leading position, with the spread of advanced technologies and the increased support of national policies, the level of ULUE in the four regions has shown a continuous convergence. In particular, from 2010 to 2016, the ULUE value of the western region has the largest change with an increase of 16.12%. The average annual growth rate of ULUE in the eastern, central, western and northeastern regions is 2.02%, 2.31%, 2.52%, and 1.89% respectively.

**Figure 4:**
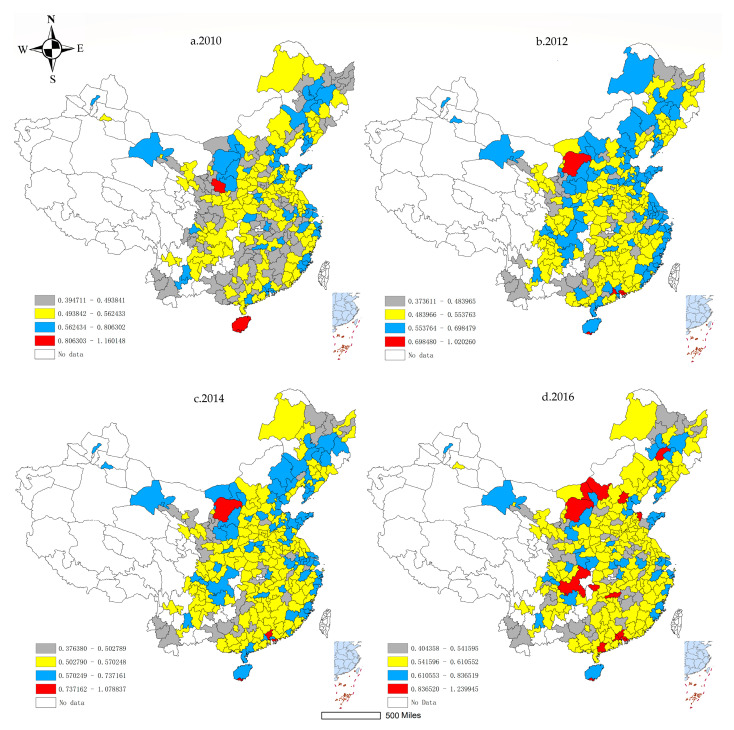
Spatial distribution of ULUE in China. (**a**–**d**) are spatial distribution maps of ULUE in 2010, 2012, 2014, and 2016.

To understand the dynamic spatial pattern of ULUE change in China, the ULUE in 2010, 2012, 2014 and 2016 are visualized using the ArcGIS 10.2 software (Esri Inc, Redlands, CA, USA) (Figure 4). 

Overall, the ULUE shows obvious spatial differences. The areas with high ULUE cover the major eastern coastal developed cities and a few western cities. The Pearl River Delta, Yangtze River Delta, and Beijing-Tianjin-Hebei regions enjoyed the higher ULUEs. From the results of four-time nodes selected, the ULUE of each region has the characteristics of continuous dynamic change in space, among which Chengdu-Chongqing urban agglomeration has the most significant increase, while the eastern coastal urban agglomeration has the obvious trend of point-to-plane expansion growth. The reason for the regional spatial differences of ULUE may be that the eastern coastal areas have innate geographical advantages, and the Chinese government have given it the earliest and vigorous policy support. The eastern region has a friendly business environment and comprehensive industrial support facilities, which has realized the industrial transformation and upgrading from traditional economy to high-tech economy earlier. The economic development levels in the central and western regions are relatively backward, and still relying on the development of traditional manufacturing. Although the current regional industrial relocation from eastern has brought huge economic benefits, but at the cost of natural resources and brings in high undesirable outputs, thus affecting the improvement of ULUE. The northeastern region used to be the most important heavy industry base in China, but due to its relatively homogeneous industrial structure, it was soon at a disadvantageous in the market competition. Although the China government has issued several policies aimed at revitalizing the economy of the northeastern region, subject to the path dependence of its development path and brain drain, the improvement of ULUE value in the northeastern region is still the slowest among the four regions.

#### 4.1.2. Input Redundancy Analysis of Non-DEA Effective DMU

**Figure 5:**
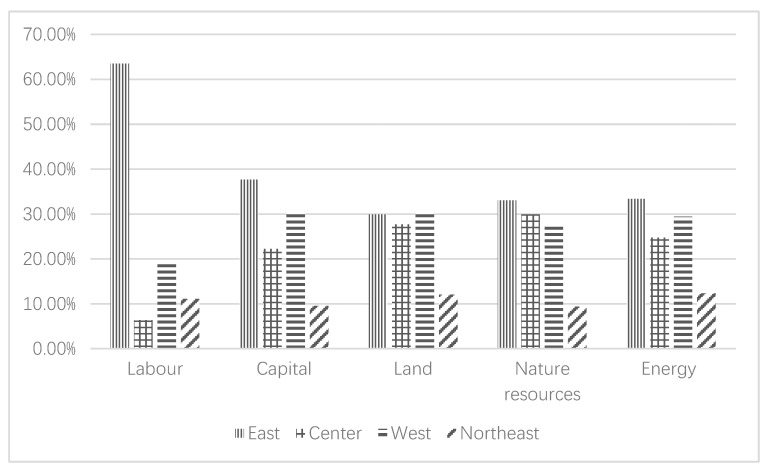
Input redundancy of DMUs.

The economic significance of ULUE obtained under the condition of constant return to scale refers to the actual ratio of input required when the output level remains unchanged and the best-performing DMU is used as the standard. Based on this, the calculation results of redundancy value of each input element are shown in Figure 5. 

(1) The 63 DMUs that need to be reduced in labor input are mainly distributed in the eastern region, most of which belong to large cities and megacities. (2) There are 984 DMUs to be reduced in capital input, of which the proportions in the eastern, central, western and northeastern regions are 37.7%, 22.3%, 30.5% and 9.6%, respectively. (3) The number of DMUs that need to be reduced for urban construction land use reached 1974, accounting for 98.26% of the total DMU, indicating that during the study period, most Chinese cities had extensive land use. (4) The number of DMUs with redundant natural resources input is 1667, of which the ratios of redundant DMUs in the eastern and central regions to the total number of DMUs in their respective regions reached 90.5% and 89.1%, indicating that these two regions over-reliance on the depletion of natural resources in the process of socioeconomic development. (5) The number of DMUs needed to reduce energy input is 1497, mainly in the eastern and western regions. It can be seen from the above results that a large number of DMUs with redundant input factors are mainly reflected in the relaxation of multiple input factors such as land, natural resources and energy, which ultimately leads to a lower ULUE value.

### 4.2. Spatial Econometric Analysis of ULUE

#### 4.2.1. Spatial Correlation Characteristics of ULUE

The global Moran’s *I* indexes of ULUE in Chinese cities from 2010 to 2016 are calculated by Stata software (StataCorp LLC, College Station, TX, USA) based on the economic-distance weight matrix. The results are shown in Table 4, from 2010 to 2016, the global Moran’s *I* indexes of ULUE in China are all positive, which have passed the statistical test of significance level with *p* value less than 0.01. These results suggest that the spatial distribution of ULUE in China is not random, but presents a certain spatial convergence feature, and has a positive spatial correlation, that is, the spatial agglomeration characteristics of high-value agglomeration and low-value agglomeration. There is a proximity effect among cities, and the ULUE level of a city depends on the ULUE level of its neighboring cities. More importantly, the spatial agglomeration pattern is relatively stable in the study time series. Hence, it is necessary to pay attention to the spatial features among regions in the study of the influencing factors of ULUE.

#### 4.2.2. Spatial Influencing Factors of ULUE

After the LM and R-LM tests, the results show that the LM and R-LM values of the SEM are significantly larger than the LM and R-LM values of the SLM, which indicates that the spatial correlation of ULUE is more focused on the spatial error effect (Table 5). Therefore, the SEM is selected to analyze the influencing factors of ULUE spatial correlation. The Hausman test results show that the P-value is less than 0.1, which indicates that the null hypothesis is rejected, so the fixed-effect model is more appropriate for parameter estimation. The results show that the fitting degree and estimation result of time fixing effect is larger than that of individual-fixed and double-fixed effect models. Therefore, the estimation results of the time-fixed effect SEM are selected to implement the empirical study of ULUE in China. 

To facilitate comparative analysis, the result of traditional panel regression is also displayed, as shown in Table 6. It can be seen that the goodness of fit of the SEM is higher than that of the ordinary panel model, which shows that the traditional linear regression model ignores the spatial influence factors, making the model not suitable, and ultimately leads to biased estimation results. Based on the regression results of the full sample model, the spatial correlation error coefficient λ value is significantly positive, which further proves the existence of the spatial dependence of ULUE in China. Among the four regional models, the results show that only the λ value in the eastern region is significant and passed the 1% significance level test, demonstrating that the spatial linkage of economic activities within the eastern region is higher than that in the central, western and northeastern regions.

From the results of the full sample model, economic agglomeration is significantly positive at the 5% level, indicating that economic agglomeration can optimize China’s ULUE. In other words, China’s ULUE is still in the stage of increasing with the escalate in the degree of economic agglomeration, and the agglomeration effect is greater than the congestion effect. Therefore, to make better use of the economic agglomeration effect, avoiding the emergence of the congestion effect should be the core content of urban land use management. However, from the results of the regional model, only the coefficient of northeastern region is significantly positive, while the coefficients of eastern, central and western regions are not significant. These results indicate that economic agglomeration does not have a close interaction with ULUE within individual regions, but it has a wider geographical space impact across regions.

The industrial structure is significantly positively correlated with ULUE in the full sample model, representing that the upgrading of industrial structure is conducive to guiding resources and elements to sectors with higher production efficiency, exerting structural leverage and improving the output level of unit land. The impact mechanism of the industrial structure on ULUE has significant regional heterogeneity. The industrial structure level is significantly positive in the eastern and central models, while the coefficients in the western and northeast models are not significant. In the western region, industries are mainly concentrated in low-end manufacturing, which is in the middle and lower reaches of the industrial chain. Deliberately increasing the proportion of the tertiary industry is not suitable for the development needs of the current western cities. Industrial structure readjustment is a process of “creation and destruction coexisting”, the over-optimistic pursuit of advanced industrial structure will weaken or even destroy the foundation of industrial rationalization, which is not conducive to the rational allocation of resource elements among industries. Compared with emerging industries such as information, consulting, technology and finance that are gradually taking the lead in the tertiary industry in the eastern and central regions, the proportion of traditional service industries such as commercial catering and transportation in the northeast and western regions is still relatively large. The tertiary industry with high efficiency, high added value, and low environmental negative effects have not yet formed scale in the western and northeastern regions, resulting in insignificant efficiency improvement.

The coefficient of economic development has a significant positive effect on ULUE in the central, western, northeastern and full-sample models. However, in the eastern region model, the level of economic development has no significant impact on ULUE. The economic development level of the east is ahead of the whole country, and the gradient of China’s economic development is gradually decreasing from east to west. The regression results provide evidence to support the Kuznets curve hypothesis of ULUE, and there is an inverted U-shaped relationship between economic development and ULUE. That is to say, with the development of the economy, the efficiency of ULUE may first increase rapidly, then slowly grow, and then the region will flatten or even decline.

From the regression results of the full sample model, the coefficient of the government intervention is significantly positive at the 1% level. However, there are some differences in the direction and degree of variable influence in the four regional models. The coefficients of the variables in the eastern and western regions are positive and significant, but in the northeastern and central model, the influence of variables is insignificant and negative, respectively. These results indicate that in the central and eastern regions, the government intervention has not reached the expected goal of policy formulation, even disturbed the normal operation of land use mechanism to a certain extent, triggered the inefficient allocation of production resources and inhibits the improvement of ULUE.

The sci & edu investment variable is significantly positively correlated with ULUE in the full sample model. The results of different regional models show that the promotion mechanism of sci & edu investment level to ULUE improvement mainly occurs in the eastern and central regions. This is mainly due to the relatively strong scientific research base and high-end talents gathered in the eastern and central regions. In contrast, most cities in the western region are still in the stage of undertaking the relocation of low-end and labor-intensive industries from the eastern coast. The development of such type of industries are relatively low in the demand for high-end science and technology. Besides, the northeastern region has experienced a large number of brain drains due to economic downturn. The impact of sci & edu investment on regional ULUE has an obvious “Matthew effect”.

In the central region model, the coefficient of environmental governance has a positive and significant impact on ULUE, but in the full sample model and the other three regional models, the impacts of environmental governance are not significant. The central region is a pilot demonstration area for China’s environmental governance, and has taken the lead in implementing the “resource-saving and environment-friendly society” policy, which reduces the ecological negative effects in the process of land use and promotes the ULUE. However, most cities in China are still in the stage of emphasizing economic construction, and environmental governance cannot alleviate the massive ecological negative effects caused by economic development. Also, improving ULUE through environmental governance is a long-term process, and various policies and measures adopted are difficult to achieve goals in the short term. As a result, the promotion mechanism of ULUE appears insignificant.

The energy consumption level has a negative impact on the ULUE in all models. Compared with developed countries, traditional fossil energy sources such as coal still occupy the dominant position of China’s energy consumption structure. Lower energy efficiency and higher total energy consumption result in more undesirable output during the production process, which ultimately leads to a decline in ULUE. From the perspective of the coefficient of energy consumption level, the negative influential intensity of energy consumption on ULUE is in the descending order of west, central, northeast and east.

From the result of the full sample model, the impact of land marketization on ULUE is not significant. It shows that the improvement of land marketization at the national level cannot effectively improve the ULUE. The results of the models in the eastern, central, western, and northeastern regions vary widely. The degree of land marketization in the eastern and central regions has no significant impacts on ULUE, while in the western and northeastern regions it has significantly positively affect ULUE at 1% and 5% level, respectively. The land market in the eastern and central regions is relatively mature, and the marginal utility of the degree of land marketization on ULUE improvement mechanisms is reduced. The western and northeastern regions are in the stage of constructing and improving the land market, and gradually begin to pay attention to the role of resource allocation through market mechanism, which is beneficial for the promotion of ULUE.

## 5. Discussion and Policy Implications

On the one hand, the improvement of ULUE can realize the sustainable urban growth and coordinate economic development and environmental protection; on the other hand, the improvement of ULUE will directly or indirectly affect the welfare and health of the public. From the perspective of natural resource input, China’s current urbanization is at the expense of conversion of high-quality agricultural land and ecological land. The high quality, safety, agricultural products, and biodiversity are neglected and sacrificed in this process, which indirectly endangers the welfare and health of the public. From the perspective of undesirable output, high economic output is accompanied by high pollution and high emissions, which directly affect public health and welfare. Hence, on the basis of the study on ULUE in this paper, we attempt to put forward the following policy implications to improve ULUE in China. First, in order to upgrade and optimize the urban industrial structure, scientific industrial policies should be formulated according to the characteristics of the city. Establishment of industrial investment funds can increase support for the development of high-end industries. Meanwhile, encouraging communication among government departments, education and research units across regions to enhance the linkage driving effects of economic development. Second, reduction in unnecessary government intervention in economic activities so as to make the market play a decisive role in resource allocation by creating a good business environment for the development of enterprises and industries. At the same time, controlling pollution from the source by tightening access for enterprises and avoiding the entry of high emission and high energy consumption enterprises. All the above efforts combining with input element saving and intensive use can realize smart urban growth. Third, it is necessary to give priority to increasing the investment in science and technology innovation and establish a cooperative system that includes enterprises, research institutions and universities. Facilitating the transition from a fossil fuel based economy to a green and circular economy that is for the largest part based on renewable resources.

However, this study has several limitations. First, we adopted only a 7-year sample period because of the unavailability of data. We will try to obtain more data to extend the study period to produce more convincing and reliable results. Second, in the process of calculating the input value of natural resources, the official data used cannot completely reflect the transformation of different types of land in Chinese cities. In fact, a large number of ecological lands have been illegally converted into construction land. Due to the lack of relevant data, this study did not include this part of illegal land conversion into the measurement system. Future research can use remote sensing image data to truly reflect this type of land use change. Third, the relationship between influencing factors such as the level of economic development and ULUE is a dynamic reciprocal interaction. However, this study only considers the mechanism of unilateral impact on ULUE. More attention should be paid to the analysis of influencing factors and ULUE, we will make improvements to these limitations in future studies. In addition, here are some horizons for ULUE research in the future for better use and manage urban land. First, from the research scale perspective, multi-scale research should be strengthened. Micro-scale research is conducive to discovering the behavior and reasons of micro-subjects and explaining the micro-mechanism of urban land use efficiency changes. Future research can explore the mechanism of urban land use changes caused by the interaction between residents, real estate developers, and governments, as well as the interaction between land users and the environment. Second, the research objects of existing ULUE studies are administrative urban areas, rather than cities in real land use patterns. Therefore, further research is needed to decompose the non-built-up areas in a city, so that the input and output conditions are closer to the real world. Third, in the context of the gradual escalation of the government’s focus on natural resources, it is necessary to consider policy factors into the consideration of ULUE’s influence factors.

## 6. Conclusions

In this paper, we conducted a case study of 287 cities in China and investigated the ULUE, its spatial correlation characteristics and influencing factors from 2010–2016 by using the super efficiency SBM model and spatial panel model. The main conclusions are as follows:

(1) The overall ULUE of Chinese cities was still at a relatively low level during the study period. The eastern region has the highest ULUE value, and the rest are in the western, central and northeast in order. From 2010 to 2016, the ULUE of various regions in China rose steadily, and the differences among regions showed a trend of continuous convergence. The ULUE in China presents significant spatial dynamic changes. The Chengdu-Chongqing urban agglomeration has the most significant increase in ULUE and the improvement of ULUE of eastern coastal urban agglomeration shows a clear trend of point-to-plane and belt-like expansion. 

(2) Almost all elements have input redundancy in the input-output process of urban economic operation. The redundancy rates of land, natural resources and energy are at a relatively high level, and that of labor is at a low level. Reducing these input redundancy rate will effectively improve the ULUE in China and achieve optimal allocation of urban land resources.

(3) China’s ULUE has a significant positive spatial correlation from 2010 to 2016. From national perspective, in addition to energy consumption, economic development, government intervention, sci & edu investment, economic agglomeration and industrial structure play important roles in the promotion of ULUE. From regional perspective, the influencing factors of ULUE are heterogeneous. The level of economic development plays an important role in improving ULUE in the central, western and northeastern regions. The government intervention in the eastern region is more effective in improving ULUE than in other three regions. Especially, government intervention has a significantly negative impact on ULUE in the northeastern region, which indicates that the administrative capacity of this region is relatively low and hinders the regional economic development. Investments in sci & edu and the industrial structure are fundamental in improving ULUE in eastern and central regions. The economic agglomeration and environmental governance will only promote the development of ULUE in the northeastern and central regions, respectively. The energy consumption is significantly negative with ULUE in the eastern, central and western regions. The land marketization has a positive impact on ULUE in the western and northeastern regions.

## Figures and Tables

**Table 1:** Input and output indicators.

Category	Indicator	Description	Units
Input	Capital	Fixed capital stock	10^8^ Yuan
	Land	Urban construction land	km^2^
	Labor	Urban employment	10^4^ Persons
	Energy	Total standard coal equivalent consumption	10^4^ Tons
	Natural resources	ecosystem service value of depleted natural resources	10^8^ Yuan
Desirable output	GDP	GDP value of secondary and tertiary industries	10^8^ Yuan
Undesirable output	Smoke	Emissions of industrial smoke	Tons
	Sewage	Emissions of industrial sewage	Tons
	SO_2_	Emissions of industrial SO_2_	Tons
	PM2.5	Average annual PM2.5 value	μg/m^3^

**Table 2:** Influencing factors of ULUE.

Variables	Description	References
Economic Agglomeration	The population density (people per square kilometer)	Guastella et al. (2017) [46]
Industrial Structure	Ratio of output value of tertiary industry to the secondary industry (%)	Chen et al. (2018) [64]
Government Intervention	Per capita financial expenditure (10^4^ yuan)	Tu et al. (2014) [65]
Economic Development	Per capita GDP (10^4^ yuan)	Gao et al. (2019) [66]
Sci & edu investment	The proportion of science and education expenditure to fiscal expenditure (%)	He et al. (2020) [67]
Environmental Governance	Comprehensive waste treatment rate (%)	Ma et al. (2019) [68]
Unit energy consumption	Unit GDP energy consumption (ton of standard coal)	Huang et al. (2020) [69]
Land Marketization	The proportion of land sold by bidding, auction and listing to the total amount of land supply (%)	Liu et al. (2016) [70]

**Table 3:** Trends of ULUE in China during 2010–2016.

Region	2010	2011	2012	2013	2014	2015	2016	Mean
China	0.528	0.532	0.544	0.549	0.557	0.566	0.603	0.554
East	0.565	0.570	0.580	0.579	0.590	0.598	0.637	0.588
Central	0.505	0.511	0.523	0.528	0.536	0.543	0.579	0.532
West	0.515	0.519	0.535	0.546	0.550	0.561	0.598	0.546
Northeast	0.521	0.520	0.528	0.534	0.536	0.550	0.583	0.539

**Table 4:** The Moran’s *I* index of ULUE in China from 2010 to 2016.

Year	Moran’s *I* index	*Z*-Value	*p* Value
2010	0.038	5.638	0.000
2011	0.076	10.820	0.000
2012	0.067	9.582	0.000
2013	0.027	4.230	0.000
2014	0.064	9.274	0.000
2015	0.060	8.554	0.000
2016	0.070	9.899	0.000

**Table 5:** LM and robust LM test results of space lag effect and space error effect.

Test	Statistics	*p* Value
LM (Lag)	8.803	0.003
Robust-LM (Lag)	1.459	0.227
LM (Error)	497.025	0.000
Robust-LM (Error)	489.682	0.000

**Table 6:** Regression results of influencing factors of ULUE.

Variable	China	China	East	Central	West	Northeast
	OLS	Time Fixed-Effect Spatial Panel Regression				
Economic Agglomeration	0.011	0.004 **	0.006	−0.003	0.003	0.013 **
Industrial structure	0.0413 ***	0.040 ***	0.072 ***	0.053 ***	−0.009	0.014
Economic development	0.0219 **	0.065 ***	0.016	0.070 ***	0.064 ***	0.121 ***
Government intervention	0.0439 ***	0.024 ***	0.045 ***	−0.008	0.025 *	−0.083 ***
Sci & edu investment	−0.0046	0.220 ***	0.277 ***	0.295 ***	−0.031	0.158
Environmental governance	−0.00243	0.002	0.056	0.039 **	−0.006	−0.011
Unit energy consumption	−0.00361	−0.014 ***	−0.015 ***	−0.019 ***	−0.027 ***	−0.012
Land marketization	0.0104	0.012	−0.027	−0.027	0.044 **	0.080 ***
λ		0.448 ***	0.303 ***	−0.064	−0.013	−0.278
R^2^	0.343	0.386	0.431	0.459	0.378	0.447
log-L		2605	702.5	1045	773.6	378.5
N	2009	2009	609	560	602	238

Note: ***, ** and * represent the significance level of 1%, 5%, and 10%, respectively.
